# Coincidence Analysis of the Cropland Distribution of Multi-Sets of Global Land Cover Products

**DOI:** 10.3390/ijerph17030707

**Published:** 2020-01-22

**Authors:** Chengpeng Zhang, Yu Ye, Xiuqi Fang, Hansunbai Li, Xue Zheng

**Affiliations:** 1 Faculty of Geographical Science, Beijing Normal University, Beijing 100875, China; cpzhang@mail.bnu.edu.cn (C.Z.); xfang@bnu.edu.cn (X.F.); lihansunbai@mail.bnu.edu.cn (H.L.); zhengxue@mail.bnu.edu.cn (X.Z.); 2 Key Laboratory of Environment Change and Natural Disaster, Ministry of Education, Beijing Normal University, Beijing 100875, China

**Keywords:** global, cropland cover, coincidence, uncertainty, multi-products, overlay

## Abstract

Modern global cropland products have been widely used to assess the impact of land use and cover change (LUCC) on carbon budgets, climate change, terrestrial ecosystems, etc. However, each product has its own uncertainty, and inconsistencies exist among different products. Understanding the reliability of these datasets is essential for knowing the uncertainties that exist in the study of global change impact forced by cropland reclamation. In this paper, we propose a set of coincidence assessments to identify where reliable cropland distribution is by overlaying ten widely used global land cover/cropland datasets around 2000 AD. A quantitative assessment for different spatial units is also performed. We further discuss the spatial distribution characteristics of different coincidence degrees and explain the reasons. The results show that the high-coincidence proportion is only 40.5% around the world, and the moderate-coincidence and low-coincidence proportion is 18.4% and 41.1%, respectively. The coincidence degrees among different continents and countries have large discrepancies. The coincidence is relatively higher in Europe, South Asia and North America, while it is very poor in Latin America and Africa. The spatial distribution of high and moderate coincidence roughly corresponds to the regions with suitable agricultural conditions and intensive reclamation. In addition to the random factors such as the product’s quality and the year it represented, the low coincidence is mainly caused by the inconsistent land cover classification systems and the recognition capability of cropland pixels with low fractions in different products.

## 1. Introduction

Since the agricultural revolution, approximately 12.2% of the world’s land area has been reclaimed as cropland [1]. Such drastic, extensive and long-lasting land use patterns have been and are continuing to have a profound impact on global change [2]. The cropland reclamation not only affects terrestrial ecosystems [3] and biodiversity [4,5] via directly reshaping the primary land cover types but also has a profound impact on regional or global change by changing both the physical conditions of the land surface (e.g., albedo, radiative forcing, and evapotranspiration) [6,7,8] and the biogeochemical cycles of carbon and nitrogen [9,10,11,12,13]. Comprehensive and accurate assessments of the cropland-caused impact of global climate change mainly depend on precise global cropland products [1,14,15,16]. Therefore, improving the quality of cropland products (e.g., spatial resolution and data accuracy) is one of the most important ways to reduce the uncertainties of the comprehension on how to affect global change [17,18,19].

Plenty sets of modern and historical global land cover products have been developed by several international research institutes and universities to meet the needs of related global change research [20,21,22]. The coverage periods of modern products have mainly focused on recent decades since the 1980s [23], and the spatial resolution has gradually improved from 1 km × 1 km to 30 m × 30 m [24,25,26], even to reach 10 m × 10 m [27]. The coverage periods of historical products have continuously extended to the Holocene [1,22] and most of them are mainly focused on the past 300 years [28,29], but their spatial resolutions are relatively coarse, mainly 0.5° × 0.5° or 5′ × 5′. Applied as input parameters for global and regional climate models, ecosystem models, carbon cycle models, these global cropland products have been widely used to assess the impact of land use and cover change (LUCC) on global and regional carbon budgets, climate change, terrestrial ecosystems, etc. [5,13,14,15,20].

The development and improvement of modern global land cover products/cropland datasets benefit from the gradual advancement of remote sensing technology and interpretation methods. So far, many modern land cover products have been created for global change research, such as IGBP-DSICover [25], GLC2000 [30], ESA-CCI-LC [20], GlobeLand30 [21], and GLC-Consensus [31]. The datasets specialized for the cropland class have also been produced, such as USGS-GFSAD [32] and HybridCropland [33]. Not only the spatial resolutions of the above products have been greatly improved, but also the interpretation accuracy of these satellite-derived Boolean products has also been significantly improved [34]. For instance, the overall classification accuracy of IGBP-DISCover (1992–1993) released in 2000 was only 66.9%, and that of GlobeLand30 (2000) released in 2015 has increased to 80.3% [34]. In addition, the synergized datasets based on the synergizing of multi-sets of Boolean products has improved the spatial accuracy, and the fractional data format is more applicable in the research of global change impact (e.g., ecological simulation) [31], making it easier to carry out quantitative assessments and comparisons of products.

Each set of Boolean products has executed a corresponding accuracy or reliability evaluation during the establishment process, and the accuracy of synergized products is also assessed by independent validation samples [31,33]. In addition, some assessment studies made comparisons among multi-sets of products that have similar time periods and in the same region [35,36,37]. However, the results of all the products’ validation or assessment studies have shown that there are still obvious uncertainties in these products [38,39,40,41]. On the one hand, there is an evitable interpretation error in each product [42,43]. Most products’ accuracies are only approximately 70% [34] and the accuracy is obviously different in different regions [30,36]. On the other hand, comparisons of the spatial distribution and amount among different satellite-derived products indicate that large inconsistencies exist at global, continental, and national scales [35,37,38,39,44,45]. Previous studies usually attribute these differences to the acquisition time of original remote sensing images and the various interpretation methods from the remote sensing perspective [34,46,47].

Analyzing the coincidence among multi-sets of global land cover products is a feasible way to evaluate the reliability of these products [39,45,48]. Although several studies of the coincidence assessment have been conducted, the evaluated datasets are mainly focused on the original satellite-derived products, and the assessment classes usually contain all the land cover types [35,36,37,38,39,40]. Only a few evaluation studies are specifically focused on the cropland type, and most of them are conducted on continental or national scales [45], global-scale evaluations should be carried out. Increasingly, synergistic products are not fully considered in assessment studies. In addition, the number of coincidence analysis products is not enough (usually less than five). Thus, a few products with poor accuracy will increase the large uncertainty of evaluation results. In addition, the goal of most assessment studies is to evaluate the reliability of individual products. Different from previous studies, we intend to get a comprehensive understanding of the consistency of cropland spatial distribution indicated by these products. When applying these cropland datasets in the fields of global change impact evaluation, spatial explicit reconstruction of historical cropland, etc., it will beneficial for getting a deep understanding of uncertainty in related researches [1,15].

According to the extensive application in previous researches, the 2000 AD. was carefully chosen as an important time section to preferentially carry out the coincidence analysis. First, whether in terms of spatial resolution, interpretation method, or data format, the products around this section are very plentiful. Second, the usage of these products around this section is also relatively broad, especially in the studies of spatial explicit reconstruction of historical cultivation. Since this section can generally reflect the maximum cropland reclamation in the modern era than other decades on a global scale [22,29]. In this paper, we collected ten widely used global land cover products or synergizing datasets around 2000 AD. and extracted information on cropland spatial distributions. Then, the result of the cropland distribution’s coincidence at the pixel scale was generated according to the overlay process of multi-sets of cropland subsets. We aimed to identify the locations where cropland was reliable and unreliable. Namely, we assumed the coincidence degree as a criterion for judging the reliability of cropland distribution represented by multi-sets of datasets. Moreover, we further calculated the percentage of different reliabilities in different spatial units based on the overlay result of the coincidence degree. Finally, we discussed the spatial distribution characteristics of cropland with different coincidence degrees and further explained the main reasons for such spatial differentiation of coincidence.

## 2. Materials and Methods

### 2.1. Data Sources

The cropland datasets used in this study were derived from ten global land cover or cropland products, including seven satellite-derived Boolean products and three synergized fractional datasets. The spatial resolutions all of these products reached 1 km × 1 km, and the years represented by these products are mainly around 2000 AD. The seven Boolean datasets can be classified into three categories according to the land cover classification systems (LCCSs) they adopted: IGBP-DISCover [25], GLC-UMD [49], and GLC-MODIS [50] adopted the IGBP-LCCS and its modified systems; GLC2000 [30], GLCNMO [51], and ESA-CCI_LC [20] used the FAO-LCCS and its modified systems; and GlobeLand30 [21] adopted an independent classification system. There are some discrepancies in the cropland definitions in different LCCSs. First, the cropland defined in the GlobeLand30 is different from the former two LCCSs. The artificial pasture land and fruit gardens were classified as cropland in GlobeLand30. Second, even in the same LCCS, the same cropland category was also labeled with a different cropland fraction. For example, the cropland class in GLC-UMD is defined as a cropland fraction over 80% in per-pixel, while it was only over 60% in IGBP-DISCover. In addition, the low fraction (less than 10% or 15%) cropland pixel cannot be interpreted in the Boolean products at 1 km resolution, but it can be easily identified in fractional format datasets. The three fusion datasets based on the synergizing of multi-sets of global land cover products are GLC-Consensus [31], GLC-Share [19], and HybridCropland [33]. The total amount of cropland has been further calibrated by national or subnational statistical data in the latter two datasets. The detailed information of different LCCSs and the interpretation methods adopted by these products can be found in the referenced articles. The cropland distribution around 2000 AD. was represented by these products with various interpretations and preparation methods, multi-resolutions, and different formats. The cropland-related information of the ten products used in this study is shown in Table 1, including the product name, overall accuracy, spatial resolution, year represented by the product, code of cropland classes in its LCCS, and the corresponding inferred cropland proportional range. It is worth noting that the overall accuracy of the most Boolean products is only about 70%, and the accuracy of fusion datasets has been increased to over 80%. However, the cropland distribution pattern indicated by the original satellite-derived products had been slightly changed in these fusion datasets due to the synergizing and quantitatively calibration process. The accuracy of some fusion fractional datasets is not as high as the publisher claimed [52].

Although the year represented by IGBP-DISCover and GLC-UMD is a little far from the 2000 AD, these products were widely used in related researches and were absorbed in the above-mentioned fusion cropland datasets. Therefore, these two products still participated in the coincidence analysis by tolerating this defect.

### 2.2. Methods

The method we proposed in this paper to assess the coincidence of cropland spatial distributions among these different products is focused on the circumstance that there are plenty of products, but they lack an objective reference dataset for accuracy assessment. This method assumes that the original satellite images and interpretation methods adopted in each product have their own rationality and reliability (e.g., each product executes its own accuracy validation) [53]. However, there are still certain errors and uncertainties. This means that a considerable proportion (usually more than 60%) of the pixels in each product can correctly reflect the real geographical surface with corresponding land cover types, while there are also a small number of misjudged pixels that are inconsistent with the real world.

We extracted the cropland-related classes in each product and consider it as an expert judgment on the spatial distribution of cropland. Then, the coincidence degree of cropland spatial distribution was generated based on overlaying these cropland datasets. Namely, at a certain pixel, how many products identified it as cropland class? When all products agree that a certain pixel is of cropland class, this pixel has the highest coincidence; when no one product identifies a certain pixel as cropland, this pixel has the lowest coincidence. The coincidence result was used as an index to assess the reliability of cropland identified at the pixel scale. There might be a slight risk when using the coincidence degree as a criterion to evaluate the reliability of cropland spatial distribution. It cannot be completely identified as cropland even the pixels with the highest degree of coincidence, because of the few inevitable defects in the satellite image interpretation process, such as: the same object with different spectrums or same spectrum with different objects. However, from the application view, when a certain pixel was identified as cropland by all of the products, the uncertainty caused by the unreliability of cropland products no longer exists. Therefore, from the application perspective, we could use the coincidence degree to approximately reflect the reliability of the cropland distribution. The details of these processes are described in the following subsections.

#### 2.2.1. Data Preprocessing

We resampled the five Boolean-type global land cover products with moderate resolution into 30′′ × 30′′ (approximately 1 km × 1 km at the equator) by using the resample tool in ArcGIS (we chose WGS84 as the geographic coordinate system; the resample method was the nearest neighbor algorithm). Second, the cropland subsets were extracted from these Boolean products by selecting the cropland-related classes (cropland class and mixed-cropland class) directly. For GlobeLand30 and ESA-CCI-LC, we generated the 1 km × 1 km fishnet for the whole world land area and converted the original Boolean products to the upscaling results with 30′′ × 30′′ resolution by using the zonal statistics tool in ArcGIS instead of resampling into 1 km × 1 km directly [52]. The newly generated results are fractional type. Together with the other three synergized datasets, we masked out the noncropland pixels (the cropland fraction is 0% in pixels) and generated the cropland subsets from these five fractional datasets.

Before developing the up-scaled cropland fractional result from GlobeLand30, we first converted the original UTM projected coordinate system into an Albers projection for all 853 scene images. Then, all these images were mosaicked to two parts according to the North and South Hemispheres separately. Considering the large amount of data stored with a 30 m resolution at the global scale, the new result’s resolution in our study was adjusted to 50 m × 50 m (resampled by using the nearest neighbor algorithm). For ESA-CCI-LC, the upscaling method was similar to GlobeLand30’s method. However, unlike the 30 m resolution dataset, with the cropland class represented by pixels with a 300 m resolution, it is always difficult to reach 100% cropland. The cropland in this Boolean product is represented by several cropland-related classes with different proportional ranges. Thus, we should first set the exact cropland fraction for each cropland-related class in this product. We used the mean value instead to set the original proportional range for each class: 95% for classes 10, 11, 12, and 20; 75% for class 30; and 35% for class 40. Taking GlobeLand30 as an example, the formula for making upscaling fractional cropland results is shown as follows:

(1) $${\mathrm{Ratio}}_{1\mathrm{km}}=\left(\sum_{i=1}^{i}{\mathrm{Ratio}}_{50m}\times {\mathrm{GridArea}}_{50m}\right)/{\mathrm{GridArea}}_{1\mathrm{km}},$$

where Ratio_1km_ is the cropland proportion in the up-scaled result’s pixels with 1 km × 1 km resolution and ranges from 0% to 100%; i is the pixel number of 50 m resolution pixels in each 1 km grid; Ratio_50m_ is the cropland proportion in the original GlobeLand30 pixels with 50 m × 50 m resolution and is 100%; GridArea_50m_ is the real area of each pixel with 50 m × 50 m resolution; and GridArea_1km_ is the real area of each pixel with 1 km × 1 km resolution.

#### 2.2.2. Coincidence Assessment of the Cropland Spatial Distribution by Overlaying 10 Cropland Subsets

First, we assigned unique values to cropland pixels and noncropland pixels in 10 cropland subsets by using the raster calculator tool in ArcGIS. We assigned 1 to cropland pixels and assigned 0 to noncropland pixels. For the Boolean type result, the cropland classes and the mixed-cropland classes are coded with 1, and the rest of the land cover classes are coded with 0. For the fractional result, the pixels with noncropland are marked with 0, and other pixels are marked with 1. Then, we overlaid the ten recoded results directly and generated the coincidence result of cropland spatial distributions. The value in the result ranged from 0 to 10, where 0 indicates that there is no one dataset suggesting any cropland existed in a pixel and 10 means that all the datasets agree that cropland exists in the pixel. The larger value the overlay pixel possesses, the higher likelihood the cropland exists in this pixel. Considering the total amount of products in our study, the coincidence result of these cropland subsets was subjectively divided into three levels: when more than half of the products consider a certain pixel as cropland, it is high coincidence (it means the cropland exists at the pixel is very likely); when less than one third of the products consider a certain pixel as cropland, it is low coincidence (the cropland exists at the pixel is unlikely); the rest are moderate coincidence (the cropland exists at the pixel is likely). By calculating the percentage of different coincidence levels on global, continental, subcontinental, and national scales separately, we further assessed the coincidence of cropland distribution in different spatial units.

## 3. Results

### 3.1. Analysis of Reliability of the Cropland Spatial Distribution

**Figure 1:**
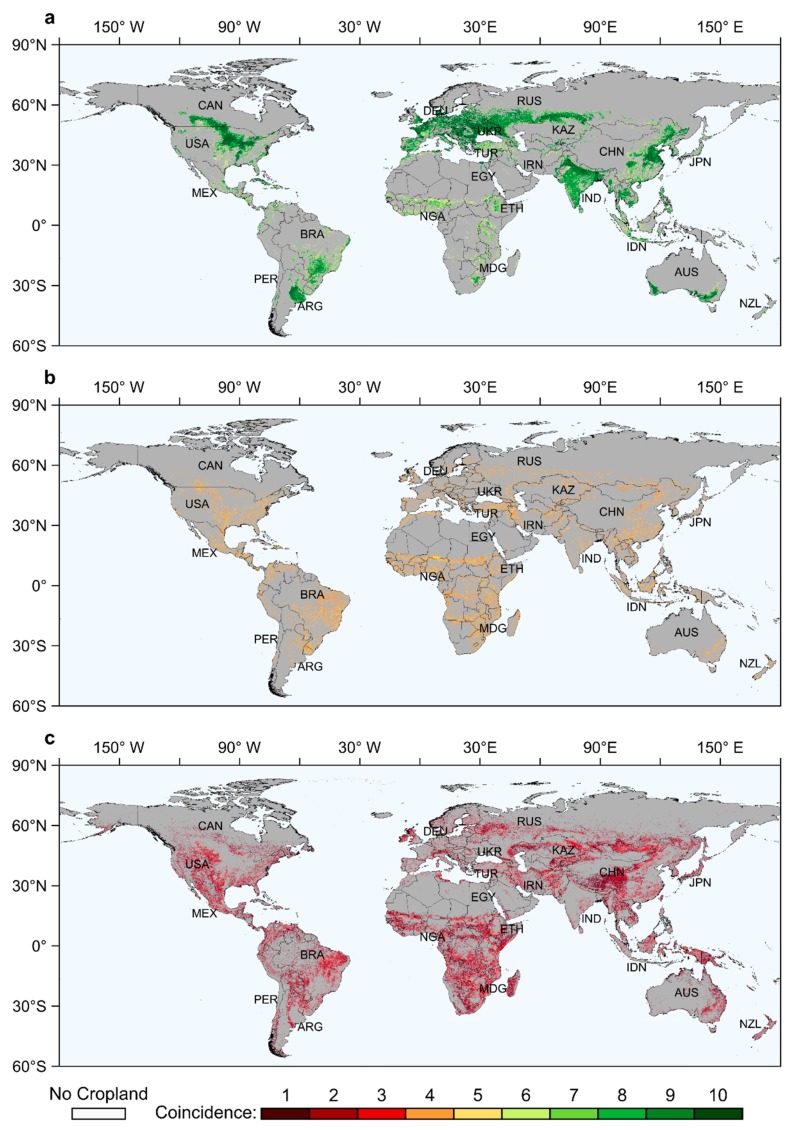
Coincidence degree of the cropland distribution around the world. The three different coincidence levels are shown separately. (**a**) is the high coincidence of cropland distribution, (**b**) is the moderate coincidence of cropland distribution, and (**c**) is the low coincidence of cropland distribution.

At the global scale, the percentages of high coincidence and low coincidence are roughly equivalent, which are 40.5% and 41.1%, respectively. The proportion of moderate coincidence is only 18.4%. Figure 1 shows the spatial distribution of cropland coincidence based on ten cropland subsets’ overlay results. It can be seen that a large amount of cropland with high coincidence is distributed in the main agricultural regions around the world (Figure 1a). These regions always have good agricultural conditions, such as India and East China in Asia; most regions in Europe (except the Nordic region); the Central Plains in North America; and Eastern Argentina and Southern Brazil in South America. The cropland with low coincidence is mainly concentrated in regions with harsh environments (Figure 1c), such as: sub-Saharan Africa, northern Europe, agro-pastoral zones and the Tibet Plateau in Asia, the large area from the northern part of the Caspian Sea extending eastward to Central Asia and Mongolia, the western part of North America, and the northeastern part of Brazil. The cropland with moderate coincidence is mainly distributed around the main agricultural regions in the form of transition zones (Figure 1b).

### 3.2. Quantitative Assessment of Reliability of the Cropland Spatial Distribution at Continental and Subcontinental Scales

**Figure A1:**
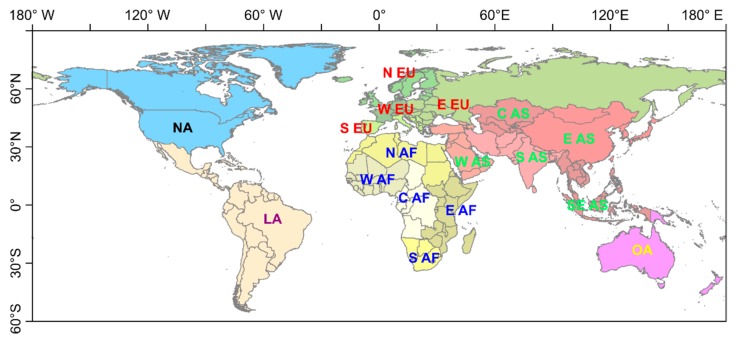
Administrative divisions adopted in this article: Asia is abbreviated as AS; Europe is abbreviated as EU; Oceania is abbreviated as OA; Africa is abbreviated as AF; North America is abbreviated as NA; and Latin America is abbreviated as LA. East is abbreviated as E; West is abbreviated as W; Central is abbreviated as C; North is abbreviated as N; and South is abbreviated as S. The original administrative boundaries and their spatial divisions are drawn according to GADM data (http://gadm.org).

We further analyzed the coincidence of the cropland spatial distribution at the continental scale (the administrative division is shown in Appendix A Figure A1) according to the sum of the percentages of different coincidence levels (in Table 2). The numerical differences between high-level coincidence and low-level coincidence in each continent are very large. The percentage of high-level coincidence in Europe (EU) is the largest, approximately 57.5%, followed by Asia (AS) and North America (NA) at 44.4% and 43.6%, respectively. The percentages in Latin America (LA) and Oceania (OA) are approximately 1/3, and Africa (AF) has the smallest proportion, which is only 22.6%. The percentage of low-level coincidence in all continents shows the relative opposite trend to that of the high levels. The percentages of low-level coincidence in Oceania and Africa are 55.5% and 51.7%, respectively; the proportions in Latin America and North America are also over 40%; Europe has the smallest proportion, reaching 30.3%. The percentage of moderate-level coincidence on all continents has relatively less difference at approximately 20%.

**Figure 2:**
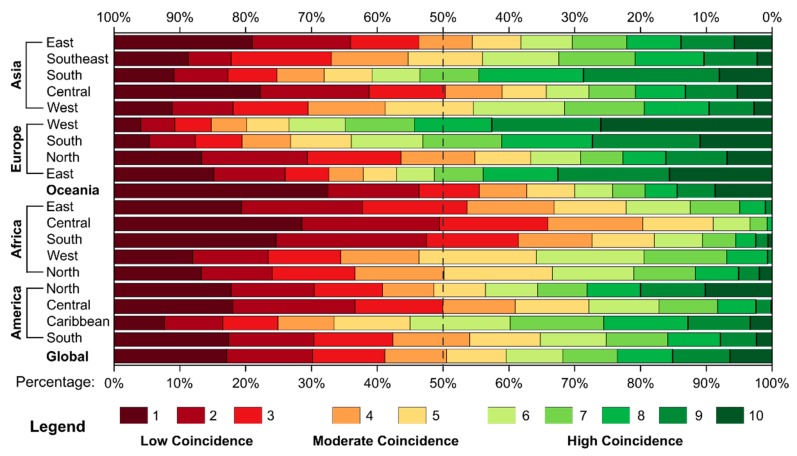
The percentage of different coincidence degrees of the cropland spatial distribution at the subcontinental scale.

We also calculated the percentages of the different degrees of coincidence of the cropland spatial distribution at the subcontinental scale (in Figure 2). The regions with over 50% of high-level coincidence included South Asia, most parts in Europe (except the Nordic region), and the Caribbean. In Western Europe, the percentage was up to 73.4%. In West Asia, East Asia, Southeast Asia, and North America, the percentages of high-level coincidence were over 40%. In Africa, only the percentage of high-level coincidence in West Africa and North Africa exceeded 30%, while those in the other regions were very small, especially in Central Africa, where the high-level coincidence was only 9.0%. In addition to the subcontinental regions mentioned above, the percentages of high-level coincidence in other regions were approximately 30%. The proportions of moderate coincidence in most regions generally ranged from 10% to 30%. From the perspective of the low-level coincidence proportion in these regions, the percentages in Central Africa, South Africa, East Africa, Oceania, and Central Asia exceeded 50%. Among these regions, the percentages of South Africa and Central Africa were extremely high at 61.4% and 65.8%, respectively. The low-level coincidence proportion of Central America was also close to 50%. The proportions in East Asia, Northern Europe, North America, and South America also exceeded 40%. The two regions with the smallest proportion of coincidence were Western Europe (14.8%) and Southern Europe (19.5%). The percentages in the rest of the regions were approximately 30%.

### 3.3. Quantitative Assessment of Reliability of the Cropland Spatial Distribution at the National Scale

**Figure 3:**
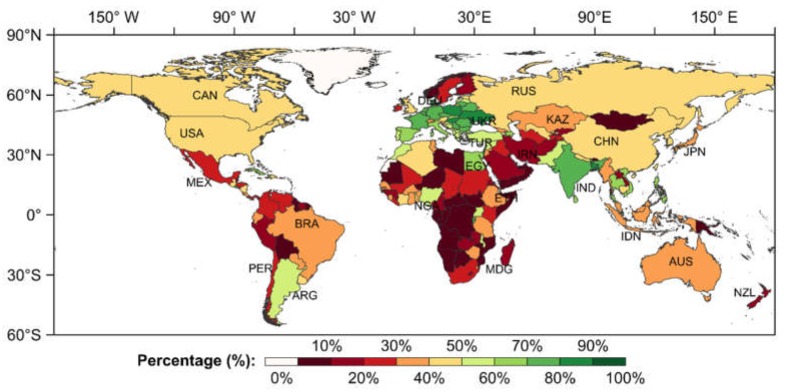
High-coincidence percentage of the cropland spatial distribution at the national scale (the exact percentage of high coincidence for each country is given in Appendix B Figure A2).

The following can be seen from the percentage of the high-coincidence cropland on a national scale around the world (in Figure 3): In Asia, the percentage of high coincidence in some Southeast Asian and South Asian countries such as Bangladesh, Thailand, and the Philippines was over 60%; the high coincidence proportion in most other countries was below 50%. As the second-largest country in Asia, China had 42.4% of cropland pixels with higher coincidence. The percentage of high coincidence in Iran, Saudi Arabia, Yemen, and some other countries in West Asia and Mongolia was commonly below 10%. In Europe, except for the four countries in Northern Europe and several countries adjacent to the Alps (e.g., Switzerland and Austria), the proportion of high coincidence in other European countries was generally more than 50%. In some Eastern European countries such as Poland and Ukraine, the proportion was over 80%. The proportion of Oceania countries was generally below 40%. In Africa, the high-coincidence percentage in most countries was generally lower than 30%, and those in some countries in Central and West Africa were even less than 10%. There were only a few countries, such as Egypt, Morocco in North Africa, and Nigeria in sub-Saharan Africa, with a percentage of over 50%. In North America, the percentages in the two countries with large territorial land areas, the United States and Canada, were slightly above 40%. The general proportion of high coincidence in South American countries was slightly higher than that in Africa. Most countries’ high-coincidence proportion ranged from 20% to 40%, and those of a few countries such as Argentina exceeded 50%.

## 4. Discussion

### 4.1. Spatial Distribution Characteristics of High and Moderate-Coincidence Cropland and the Causes

From the perspective of the cropland spatial distribution with different degrees of coincidence, cropland with high coincidence is mainly concentrated in the main agricultural regions in the world. On the one hand, due to the suitable agricultural conditions and flat terrain in these regions (e.g., the East European Plain, Great Plains in the U.S., and North China Plain) [54], the large cropland areas with continuous distribution and successive cultivation can be intensively cultivated [33]. The cropland with such features in these regions can be presented in the form of pure cropland pixels, and it is easier to be interpreted with high accuracy in various satellite images with different spatial resolutions. On the other hand, since these regions are the main food crop-producing areas (e.g., corn, wheat, and rice) [44], a few simple crop types have minimized the impact of cropland distribution discrepancies among different products caused by different cropland class definitions.

The spatial distribution of cropland with moderate coincidence mainly surrounds the cropland with high reclamation intensity, and the agricultural conditions are slightly worse and complex than in the main agriculture regions. Taking China’s agro-pastoral zones as a typical example, the cropland fraction is slightly lower than in the plain area, and this region has a large area of grassland (pasture and rangeland). These two characteristics are critical for causing the misinterpretation of land cover classes. Therefore, compared with the intensively cultivated cropland, different LCCSs, satellite image resolutions, etc., would easily reduce the coincidence degree in these regions.

Finally, the proportion of high and moderate-coincidence degrees is approximately 60%, which further proves that the accuracy of the original satellite-derived products is reliable. In these areas, using various cropland data sources would not cause significant differences in the evaluation or simulation results.

### 4.2. Spatial Distribution Characteristics of Low-Coincidence Cropland and Its Causes

We further examined the original products’ cropland classes and their reclamation intensity (cropland fractions or ranges) in the corresponding cropland distribution with low coincidence. The mosaic cropland classes in Boolean products and lower-fraction cropland pixels in fractional datasets contributed a large amount of cropland pixels with low coincidence.

In Boolean products, cropland with a moderate reclamation intensity in the real world is reflected by mixed-cropland classes. Most of these pixels are located in the regions out of the intensively cultivated area [25,30], and in such pixels, the cropland is often entangled with other land types with similar spectral characteristics [39]. Therefore, these pixels have always been identified as different land cover types with great randomness in different products [46]. Therefore, the low-coincidence cropland pixels mainly come from these cropland classes in Boolean type products.

For synergistic datasets, low-coincidence cropland pixels have a broader spatial distribution compared to the Boolean products (e.g., GLC-Consensus in the Tibet Plateau) [31]. The spatial information of synergistic datasets always inherits the spatial distribution characteristics of multi-sets of Boolean products [55]. In addition, most low-coincidence cropland is also located in regions with moderate and low cropland fractions. When assigning the exact cropland fraction to the synergized pixel, the common method that replaces the fractional ranges indicated by the original Boolean cropland classes with mean values [33,56], will further reduce the cropland fraction in some synergized cropland pixels. Therefore, a large number of low-coincidence pixels came from the fractional datasets’ cropland pixels with relatively lower fractions.

In addition, the original satellite-derived products with different spatial resolutions have apparently different capabilities for identifying cropland pixels with moderate or low fractions. That is to say, when we add the up-scaling fractional datasets derived from high-resolution products into the coincidence analysis of cropland spatial distribution, the cropland with a low fraction could easily be identified by these high-resolution products. However, pixels with low cropland fractions of less than 10% or 15% are impossible to interpret by satellite images with a moderate spatial resolution. Therefore, low-coincidence cropland will always exist in low reclamation intensity regions when making an overlay between moderate-resolution Boolean products and up-scaled results generated from high-resolution products.

Although most of the cropland pixels with low coincidence were mainly distributed in regions with low reclamation intensity, a few croplands with low coincidence could still exist in the regions with moderate or even high reclamation intensity. The explanation for the low coincidence in such regions is attributed to the different crop types (pasture, fruit trees, etc.) defined in various LCCSs by these products. For example, unlike the definitions of cropland in FAO-LCCS and IGBP-LCCS, the cropland in GlobeLand30 also includes land use types such as pasture, fruit trees, and tea trees [21,25,30]. Therefore, if there are more pastures (e.g., Central Asia and Argentina) and fruit trees or tea trees (e.g., South China) instead of pure food crops, even in the regions with high cropland fractions, low coincidence may still exist due to the different crop types contained in these products.

### 4.3. The Significance of the Application in the Field of Spatial-Explicit Reconstruction of Historical Cropland

Based on this simple method, the general recognition of cropland distribution with different reliability could be obtained directly. It is helpful for the quantitative assessment of the uncertainty among these cropland datasets. In addition, understanding the reliability and inconsistency of these basic datasets used as input for model evaluation and simulation research can further provide an insightful understanding of the uncertainty in these research results.

We will discuss the significance of the application in the study of the spatial explicit reconstruction of historical cropland. The basic assumptions in these researches are that the modern cropland distribution delineates the largest extent of historical cropland reclamation, and the difference of reclamation intensity in the modern period can also represent the historical patterns [22,29]. So, the uncertainty of modern cropland datasets directly restricts the reliability of historical reconstruction results. It is usually necessary to select or develop a set of gridded modern dataset before the allocation process. For example, Goldewijk had synergized a set of fractional cropland datasets around 2000 AD by fusing the IGBP-DISCover and GLC2000, and the historical cropland distribution was reconstructed based on this basic dataset [22]. Most previous studies focused on increasing the rationality of reconstruction by improving the allocation algorithm, but always seriously ignored the reliability evaluation of the modern input datasets they adopted [1,22,29]. In addition, the researchers often directly compared different reconstruction results in order to evaluate the reasonability of different allocation algorithms. However, ignoring these results that were generated deducted from different modern datasets is problematic. Since this study shows that the considerable inconsistency existed among the modern cropland products. Therefore, analysis of the inconsistency and the reliability among these modern input cropland products is urgently needed and is beneficial for enhancing the rationality of the historical reconstruction results, too.

### 4.4. The Uncertainty of Coincidence Analysis in this Method

In our method, to enhance the analysis of the reliability of cropland distribution based on the coincidence result requires to collect as many products around the same time period as possible. Since the time period (the 1990s) represented by the IGBP-DISCover and GLC-UMD were a little far from our target period, the uncertainty might exist in our results. Although detecting how much error these two datasets may cause in this coincidence results cannot make up the shortcoming, it is helpful for increasing the confidence of our conclusions.

We calculated the proportions of ten cropland datasets in the coincidence degree 1, respectively (in Table 3). It indicates that the IGBP-DISCover accounted for 12.4% of all the coincidence degree 1, which was apparently higher than most of the other Boolean products around 2000 AD, except the ESA-CCI-LC. The largest amount in coincidence degree 1 was contributed by GLC-Consensus. In addition, although the time period of the GLC-NMO was closer to 2000 AD than the IGBP-DISCover’s, the proportion in GLC-NMO was the second largest among these datasets. Therefore, we had to admit that the three datasets a little far from the 2000 AD contributed more than 30% of the coincidence degree 1. However, it is important to emphasize that the uncertainty of the satellite images interpretation process is mainly located in these regions with extremely low coincidence. That is to say, the uncertainty contributed by the year differences in our study will be reduced to some degree.

In addition, the number of products adopted in this study and their quality also has a slight impact on the coincidence analysis results. In terms of the absolute amount, increasing the number of products would not apparently change the result of the high and moderate coincidence parts, but the relative obvious impact on the amount of low coincidence. For example, the low coincidence proportion obviously increased when adding the GLC-Consensus into our analysis.

## 5. Conclusions

According to the analyses of cropland subsets’ overlay results derived from ten global land cover or cropland datasets that we calculated with different spatial scales, the main conclusions are as follows:

(1) The proportions of cropland pixels with high and low coincidence around the world were roughly equivalent at 40.5% and 41.1%, respectively. The proportion of moderate coincidence was only 18.4%. Most of the cropland with high coincidence was concentrated in the main agricultural regions with high cropland fraction. The cropland with moderate coincidence was mainly distributed around the main farming regions in the form of a transition zone with a moderate fraction. The cropland with poor coincidence was mainly located in regions with relatively harsh natural environments, and the cropland fraction was also relatively low.
(2) At the continental scale, Europe had the largest proportion of high-coincidence cropland (57.5%), followed by Asia (44.5%), North America (43.6%), Latin America (34.5%), and Oceania (30.1%); the proportion in Africa was the smallest, only 22.7%. The proportion of poor-coincidence cropland in Oceania was the largest (55.5%), followed by Africa (51.7%), Latin America (43.2%), North America (40.8%), and Asia (38.4%), and it was lowest in Europe (30.4%). The proportion of moderate-coincidence cropland on all continents was roughly equal, approximately 20%.
(3) At the subcontinental scale, the proportion of high coincidence in South Asia and most European regions (except for North Europe) had reached 50%; it performed the worst in central Africa, only 9.0%; the proportions in the other subcontinental regions were usually approximately 30%. The regions with a proportion of poor coincidence over 50% were West Asia and all subcontinental regions in Africa; those of most other regions were approximately 30%–40%, and Western Europe had the smallest proportion, only 14.8%.
(4) At the national scale: the proportion of high coincidence in the countries that had vast land areas and complex agricultural conditions (such as Russia, the United States, and China) was always less than 50%, except for India. The countries with moderate total cropland amount and good agricultural conditions, such as Poland and Ukraine, had the highest proportion of high coincidence, which exceeded 80%. The high coincidence in most countries in West Asia, sub-Saharan Africa, and Northern Europe with relatively harsh agricultural conditions was generally less than 20%.
(5) The spatial distribution of high and moderate coincidence roughly corresponded to the regions with suitable agricultural conditions and intensive reclamation. In addition to the random factors such as the product’s quality and the year it represented, the low coincidence was mainly caused by the inconsistent land cover classification systems and the recognition capability of cropland pixels with low fractions in different products.

In conclusion, this study was meaningful for understanding the uncertainty in the assessment of global change impact caused by cropland reclamation, and in the spatial explicit reconstruction of historical cropland, etc.

## Figures and Tables

**Table 1:** The cropland-related information in 10 global land cover products/synergized datasets.

Product	Accuracy	Resolution	Year	Cropland Classes (Boolean/Fraction %)
IGBP-DISCover	66.9%	1 km	1992–1993	12. Croplands (Boolean: 61–100)
				14. Cropland/Natural Vegetation Mosaics (Boolean: 11–60)
				Other classes (Boolean: 0–10)
GLC-UMD	65.0%	1 km	1992–1993	11. Croplands (Boolean: 81–100)
				Other classes (Boolean: 0–80)
GLC-MODIS	71.6%	1 km	2001	12. Croplands (Boolean: 61–100)
				14. Cropland/Natural Vegetation Mosaics (Boolean: 11–60)
				Other classes (Boolean: 0–10)
GLC2000	68.6%	1 km	2000	16. Cultivated and managed areas (Boolean: 61–100)
				17. Mosaic: Cropland/Tree Cover/Other natural vegetation (Boolean: 16–60)
				18. Mosaic: Cropland/Shrub and/or grass cover (Boolean: 16–60)
				Other classes (Boolean:0–15)
GLCNMO	77.9%	500 m	2003	11. Cropland (Boolean: 61–100)
				12. Paddy field (Boolean: 61–100)
				13. Cropland/other vegetation mosaic (Boolean: 16–60)
				Other classes (Boolean:0–15)
ESA-CCI-LC	71.5%	300 m	2000	10. Cropland, rainfed (Boolean: 100)
				11. Herbaceous cover (Boolean: 100)
				12. Tree or shrub cover (Boolean: 100)
				20. Cropland, irrigated or post flooding (Boolean: 100)
				30. Mosaic cropland/natural vegetation (Boolean: 71–100)
				40. Mosaic natural vegetation/cropland (Boolean: 11–50)
GlobeLand30	80.3%	30 m	2000	10. Cropland (Boolean: 100)
Hybrid Cropland	82.8%	1 km	around 2000	(Fractional: 0–100)
GLC-Share	80.2%	1 km	around 2000	2. Cropland (Fractional: 0–100)
GLC-Consensus	-	1 km	around 2000	7. Cultivated and managed vegetation (Fractional: 0–100)

**Table 2:** The percentages of coincidence levels of the cropland spatial distribution on six continents.

Coincidence	AS	EU	OA	AF	NA	LA
Low	38.4%	30.3%	55.5%	51.7%	40.8%	43.2%
Moderate	17.2%	12.2%	14.5%	25.7%	15.6%	22.3%
High	44.4%	57.5%	30.0%	22.6%	43.6%	34.5%

**Table 3:** The proportions of ten cropland datasets in the coincidence degree 1 of the overlay result.

Product	Percentage
IGBP-DISCover	12.4%
GLC-UMD	1.9%
GLC-MODIS	4.1%
GLC2000	3.3%
GLC-NMO	17.3%
ESA-CCI-LC	9.7%
GlobeLand30	3.5%
HybridCropland	4.0%
GLC-Share	5.4%
GLC-Consensus	38.4%

**Figure A2:**
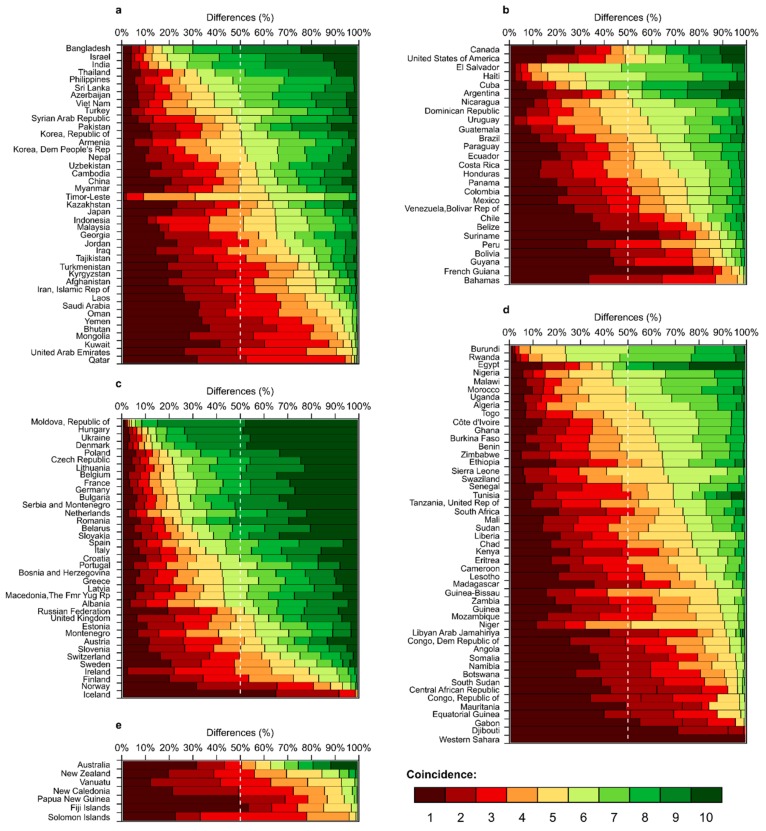
The percentages of different degrees of coincidence of the cropland spatial distribution at the national scale (the land area of country >100 km^2^), where (**a**) is Asia, (**b**) is Africa, (**c**) is the Americas, (**d**) is Europe, and (**e**) is Oceania.

## References

[1] Goldewijk K.K., Beusen A., Doelman J., Stehfest E. (2017). Anthropogenic land use estimates for the Holocene-HYDE 3.2. Earth Syst. Sci. Data.

[2] Gaillard M.J., Whitehouse N.D., Madella M., Whitehouse N. (2018). Past land-use and land-cover change: The challenge of quantification at the subcontinental to global scales. Past Land Use Land Cover.

[3] Lambin E., Meyfroidt P. (2011). Global land use change, economic globalization, and the looming land scarcity. Proc. Natl. Acad. Sci. USA.

[4] De Palma A., Sanchez-Ortiz K., Martin P.A., Chadwick A., Gilbert G., Bates A.E., Börger L., Contu S., Hill S.L., Purvis A. (2018). Challenges with Inferring How Land-Use Affects Terrestrial Biodiversity: Study Design, Time, Space And Synthesis. Advances in Ecological Research.

[5] Lanz B., Dietz S., Swanson T. (2018). The expansion of modern agriculture and global biodiversity decline: An integrated assessment. Ecol. Econ..

[6] Lambin E.F., Geist H.J. (2008). Land-Use and Land-Cover Change: Local Processes and Global Impacts.

[7] Barnes C., Roy D. (2008). Radiative forcing over the conterminous United States due to contemporary land cover land use albedo change. Geophys. Res. Lett..

[8] Vautard R., Cattiaux J., Yiou P., Thépaut J., Ciais P. (2010). Northern Hemisphere atmospheric stilling partly attributed to an increase in surface roughness. Nat. Geosci..

[9] Houghton R.A., Hobbie J.E., Melillo J.M., Moore B., Peterson B.J., Shaver G.R., Woodwell G.M. (1983). Changes in the Carbon Content of Terrestrial Biota and Soils between 1860 and 1980: A Net Release of CO_2_ to the Atmosphere. Ecol. Monogr..

[10] Matthews H.D., Weaver A.J., Meissner K.J., Gillett N.P., Eby M. (2004). Natural and anthropogenic climate change: Incorporating historical land cover change, vegetation dynamics and the global carbon cycle. Clim. Dynam..

[11] Gruber N., Galloway J.N. (2008). An Earth-system perspective of the global nitrogen cycle. Nature.

[12] Bouwman L., Goldewijk K.K., Van Der Hoek K.W., Beusen A.H.W., Van Vuuren D.P., Willems J., Rufino M.C., Stehfest E. (2013). Exploring global changes in nitrogen and phosphorus cycles in agriculture induced by livestock production over the 1900–2050 period. Proc. Natl. Acad. Sci. USA.

[13] Fuchs R., Schulp C.J., Hengeveld G.M., Verburg P.H., Clevers J.G., Schelhaas M.J., Herold M. (2016). Assessing the influence of historic net and gross land changes on the carbon fluxes of Europe. Glob. Chang. Biol..

[14] Ge Q.S., Dai J.H., He F.N., Pan Y., Wang M.M. (2008). Land use changes and their relations with carbon cycles over the past 300a in China. Sci. China Ser. D.

[15] Li B.B., Fang X.Q., Ye Y., Zhang X.Z. (2014). Carbon emissions induced by cropland expansion in Northeast China during the past 300 years. Sci. China Ser. D.

[16] Estes L., Chen P., Debats S., Evans T., Ferreira S., Kuemmerle T., Ragazzo G., Sheffield J., Wolf A., Wood E. (2018). A large-Area, spatially continuous assessment of land cover map error and its impact on downstream analyses. Glob. Chang. Biol..

[17] Verburg P.H., Neumann K., Nol L. (2011). Challenges in using land use and land cover data for global change studies. Glob. Chang. Biol..

[18] Fritz S., See L., You L., Justice C., Becker-Reshef I., Bydekerke L., Cumani R., Defourny P., Erb K., Foley J. (2013). The need for improved maps of global cropland. Eos Trans. Am. Geophys. Union.

[19] Latham J., Cumani R., Rosati I., Bloise M. (2014). Global Land Cover Share (GLC-SHARE) Database Beta-Release Version 1.0-2014.

[20] Bontemps S., Defourny P., Radoux J., Van Bogaert E., Lamarche C., Achard F., Mayaux P., Boettcher M., Brockmann C., Kirches G. Consistent Global Land Cover Maps for Climate Modelling Communities: Current Achievements of the ESA’s Land Cover CCI. Proceedings of the ESA Living Planet Symposium.

[21] Chen J., Chen J., Liao A., Cao X., Chen L., Chen X., He C., Han G., Peng S., Lu M. (2015). Global land cover mapping at 30 m resolution: A POK-based operational approach. ISPRS J. Photogramm..

[22] Goldewijk K.K., Beusen A., van Drecht G., De Vos M. (2011). The HYDE 3.1 spatially explicit database of human induced land use change over the past 12,000 years. Global Ecol. Biogeogr..

[23] Matthews E. (1983). Global vegetation and land use: New high-resolution data bases for climate studies. J. Clim. Appl. Meteorol..

[24] De Fries R.S., Hansen M., Townshend J.R.G., Sohlberg R. (1998). Global land cover classifications at 8 km spatial resolution: The use of training data derived from Landsat imagery in decision tree classifiers. Int. J. Remote Sens..

[25] Loveland T.R., Reed B.C., Brown J.F., Ohlen D.O., Zhu Z., Yang L., Merchant J.W. (2000). Development of a global land cover characteristics database and IGBP DISCover from 1 km AVHRR data. Int. J. Remote Sens..

[26] Yu L., Wang J., Clinton N., Xin Q., Zhong L., Chen Y., Gong P. (2013). FROM-GC: 30 m global cropland extent derived through multisource data integration. Int. J. Digit. Earth.

[27] Gong P., Liu H., Zhang M., Li C., Wang J., Huang H., Chen B., Clinton N., Ji L., Li W. (2019). Stable classification with limited sample: Transferring a 30-m resolution sample set collected in 2015 to mapping 10-m resolution global land cover in 2017. Sci. Bull..

[28] Ramankutty N., Foley J. (1999). Estimating historical changes in global land cover: Croplands from 1700 to 1992. Glob. Biogeochem. Cycles.

[29] Li S.C., He F.N., Zhang X.Z. (2016). A spatially explicit reconstruction of cropland cover in China from 1661 to 1996. Reg. Environ. Chang..

[30] Bartholomé E., Belward A.S. (2005). GLC2000: A new approach to global land cover mapping from Earth observation data. Int. J. Remote Sens..

[31] Tuanmu M.N., Jetz W. (2014). A global 1-km consensus land-cover product for biodiversity and ecosystem modelling. Glob. Ecol. Biogeogr..

[32] Yadav K., Congalton R. (2018). Accuracy assessment of global food security-support analysis data (GFSAD) cropland extent maps produced at three different spatial resolutions. Remote Sens..

[33] Fritz S., See L., McCallum I., You L., Bun A., Moltchanova E., Havlik P. (2015). Mapping global cropland and field size. Glob. Chang. Biol..

[34] Grekousis G., Mountrakis G., Kavouras M. (2015). An overview of 21 global and 43 regional land-cover mapping products. Int. J. Remote Sens..

[35] McCallum I., Obersteiner M., Nilsson S., Shvidenko A. (2006). A spatial comparison of four satellite derived 1 km global land cover datasets. Int. J. Appl. Earth Obs..

[36] Ran Y., Li X., Lu L. (2010). Evaluation of four remote sensing based land cover products over China. Int. J. Remote Sens..

[37] Pérez-Hoyos A., Rembold F., Kerdiles H., Gallego J. (2017). Comparison of global land cover datasets for cropland monitoring. Remote Sens..

[38] Giri C., Zhu Z., Reed B. (2005). A comparative analysis of the Global Land Cover 2000 and MODIS land cover data sets. Remote Sens. Environ..

[39] Tchuenté A., Roujean J., De Jong S. (2010). Comparison and relative quality assessment of the GLC2000, GLOBCOVER, MODIS and ECOCLIMAP land cover data sets at the African continental scale. Int. J. Appl. Earth Obs..

[40] Yang Y.K., Xiao P.F., Feng X.Z., Li H.X. (2017). Accuracy assessment of seven global land cover datasets over China. ISPRS J. Photogramm..

[41] Samasse K., Hanan N., Tappan G., Diallo Y. (2018). Assessing Cropland Area in West Africa for Agricultural Yield Analysis. Remote Sens..

[42] Pal M., Mather P.M. (2003). An assessment of the effectiveness of decision tree methods for land cover classification. Remote Sens. Environ..

[43] Phiri D., Morgenroth J. (2017). Developments in Landsat land cover classification methods: A review. Remote Sens..

[44] Pittman K., Hansen M., Becker-Reshef I., Potapov P.V., Justice C.O. (2010). Estimating global cropland extent with multi-year MODIS data. Remote Sens..

[45] Lu M., Wu W., Zhang L., Liao A., Peng S., Tang H. (2016). A comparative analysis of five global cropland datasets in China. Sci. China Ser. D.

[46] Herold M., Mayaux P., Woodcock C.E., Baccini A., Schmullius C. (2008). Some challenges in global land cover mapping: An assessment of agreement and accuracy in existing 1 km datasets. Remote Sens. Environ..

[47] Gómez C., White J., Wulder M. (2016). Optical remotely sensed time series data for land cover classification: A review. ISPRS J. Photogramm..

[48] Fritz S., See L. (2008). Identifying and quantifying uncertainty and spatial disagreement in the comparison of Global Land Cover for different applications. Glob. Chang. Biol..

[49] Hansen M.C., DeFries R.S., Townshend J.R., Sohlberg R. (2000). Global land cover classification at 1 km spatial resolution using a classification tree approach. Int. J. Remote Sens..

[50] Friedl M.A., McIver D.K., Hodges J.C., Zhang X.Y., Muchoney D., Strahler A.H., Woodcock C.E., Gopal S., Schneider A., Cooper A. (2002). Global land cover mapping from MODIS: Algorithms and early results. Remote Sens. Environ..

[51] Tateishi R., Uriyangqai B., Al-Bilbisi H., Ghar M.A., Tsend-Ayush J., Kobayashi T., Enkhzaya T. (2011). Production of global land cover data-GLCNMO. Int. J. Digit. Earth.

[52] Zhang C.P., Ye Y., Fang X.Q., Li H.S.B., Wei X.Q. (2019). Synergistic Modern Global 1 Km Cropland Dataset Derived from Multi-Sets of Land Cover Products. Remote Sens..

[53] Fang X.Q., Zhao W.Y., Zhang C.P., Zhang D.Y., Wei X.Q., Qiu W.L., Ye Y. (2019). Methodology for credibility assessment of historical global LUCC datasets. Sci. China Ser. D.

[54] Whittlesey D. (1936). Major agricultural regions of the earth. Ann. Assoc. Am. Geogr..

[55] Tsendbazar N.E., de Bruin S., Herold M. (2017). Integrating global land cover datasets for deriving user-specific maps. Int. J. Digit. Earth.

[56] Lu M., Wu W., You L., Chen D., Zhang L., Yang P., Tang H. (2017). A synergy cropland of china by fusing multiple existing maps and statistics. Sensors.

